# Alcohol Use Disorder Increases Risk of Traumatic Brain Injury-Related Hospitalization: Insights From 3.8 Million Children and Adolescent Inpatients

**DOI:** 10.7759/cureus.8740

**Published:** 2020-06-21

**Authors:** Noha Eskander, Shruti Prabhudesai, Hira Imran, Ozge Ceren Amuk, Rikinkumar S Patel

**Affiliations:** 1 Psychiatry, California Institute of Behavioral Neurosciences & Psychology, Fairfield, USA; 2 Psychiatry, Rajarshi Chhatrapati Shahu Maharaj Government Medical College, Kolhapur, IND; 3 Medicine, Rawalpindi Medical University, Rawalpindi, PAK; 4 Psychiatry, Koç University School of Medicine, Istanbul, TUR; 5 Psychiatry, Griffin Memorial Hospital, Norman, USA

**Keywords:** alcohol use, alcohol dependence, alcohol misuse, traumatic brain injury, concussion, substance use, children

## Abstract

Objectives

We conducted a cross-sectional study to identify the demographic predictors of traumatic brain injury (TBI), and the risk of association of psychiatric comorbidities including alcohol use disorder (AUD) and TBI-related hospitalizations in the children and adolescent population.

Methods

We included 3,825,523 children and adolescent inpatients (age 8-18 years) using the nationwide inpatient sample (NIS) database (2010-2014), and 61,948 inpatients had a primary diagnosis of TBI. These inpatients were grouped by comorbid AUD (N = 2,644). Multivariable logistic regression model adjusted for demographics, and psychiatric comorbidities including other substance use disorders (SUDs) was used to evaluate the odds ratio (OR) of AUD as a risk factor for TBI-related hospitalization.

Results

The majority of the TBI inpatients were adolescents (12-18 years, 82.2%), males (71.2%), and whites (59.2%). Males had three times higher odds (95% CI 3.14-3.26) for TBI-related hospitalization compared to females. Among psychiatric comorbidities, mood (4.1%) and anxiety (2.2%) disorders were prevalent in TBI inpatients, and were not associated with increased odds for TBI-related hospitalization. Among SUD, alcohol and tobacco use (4.4% each), and cannabis use (3.5%) were prevalent, and among all substances, AUD was associated with higher odds (OR 3.5, 95% CI 3.35-3.67) of TBI-related hospitalization. These patients with TBI and comorbid AUD also had higher odds for abusing stimulants (OR 5.11, 95% CI 3.85-6.77), cannabis (OR 4.69, 95% CI 4.12-5.34), and tobacco (OR 3.77, 95% CI 3.34-4.27).

Conclusion

AUD is an independent risk factor for TBI-related hospitalization with an increased risk of 50% in the children and adolescent population compared to non-alcohol users. TBI inpatients with AUD are prevalent in white, and male adolescents. These at-risk populations are also at higher risk of comorbid mood disorders and increased substance use including stimulants, cannabis, and tobacco.

## Introduction

Traumatic brain injury (TBI) is the major cause of disability with about 145,000 children and adolescents suffer from permanent cognitive, behavioral, and physical effects. Half a million children under 14 years visit the ED due to TBI in the United States (US) [1]. The incidence of TBI is two times more common in boys and in children above 10 years. Motor vehicle accidents (MVAs) represent the leading cause of death in the age group five to 24 years, blacks present more commonly with TBI from MVA [2,3].

According to the 2018 National Survey on Drug Use and Health (NSDUH), an estimated 401,000 adolescents, that is, 1.6% of adolescents (12-17 years) had alcohol use disorder (AUD). Every year 1,825 young college students die from alcohol-related unintentional accidents [4]. AUD and TBI are bi-directionally linked, and AUD increases the risk of TBI. About 30% to 50% of patients treated for TBI were intoxicated at the time of injury. Binge alcohol drinking is a major risk factor for TBI, and vice versa there is a risk of alcohol abuse in post-TBI patients that worsens the prognosis and rehabilitation outcomes [5]. AUD leads to risk-taking behaviors including driving recklessly, which leads to increased incidence of MVA, more chances of unprovoked falls, and violence-related injuries [6].

Alcohol intoxication has a direct neurotoxic effect on the brain in patients with AUD. It causes damage to the frontal lobe, cerebellum and the limbic system. Alcohol intoxication can lead to widespread white matter cerebral atrophy due to neuronal degeneration. These brain changes lead to impairment in cognition and mood abilities such as poor judgment, a decrease in concentration, impaired emotional processing, goal-directed behavior, and balance problems [7]. TBI causes diffuse axonal injury in the white matter and cerebral hemispheres leading to a lack of coordination, dizziness, poor judgment, impaired cognition, and judgment [8]. So, the co-occurrence of TBI with AUD has augmented long-term adverse effects on the brain functions [9]. AUD can help in the short-term reduction of depressive symptoms in post-TBI patients; however, it has a long-term impact on worsening anxiety and depressive symptoms [10].

Adolescents with TBI are at high risk of developing psychiatric disorders and substance abuse. Anxiety disorders, major depressive disorder (MDD), attention deficit hyperactivity disorder (ADHD), bipolar disorder, obsessive-compulsive disorder (OCD), and post-traumatic stress disorder (PTSD) are common after TBI [11]. Comorbid psychiatric disorders and AUD after TBI have negative outcomes on cognition functions and recovery [10,12].

We conducted a cross-sectional study to identify the demographic predictors of TBI, and the risk of association of psychiatric comorbidities including AUD and TBI-related hospitalizations in the children and adolescent population. Next, we discerned the differences in demographics, psychiatric comorbidities, and substance use disorders (SUDs) in TBI inpatients with AUD.

## Materials and methods

Data source

We performed a retrospective cross-sectional analysis of the nationwide inpatient sample (NIS) database (2010 to 2014) from the healthcare cost and utilization project (HCUP) [13]. The NIS provides discharge patient data from a 20% sample of all hospitals, and when discharge weights are applied to the data, the result is a weighted estimate of the total number of hospitalizations representing the US population [13]. Diagnostic information in the NIS is detected using the International Classification of Diseases, ninth edition (ICD-9) codes [13].

Inclusion criteria and outcome variables

We included children (age 8-18 years) with a primary ICD-9 discharge diagnosis for TBI as indicated by skull fractures (codes 800-801, 803-804); intracranial injury, including concussion, contusion, laceration, and hemorrhage (850.0-854.1); or unspecified head injury (959.01). Co-diagnosis of AUD was identified using the ICD-9 codes 291.0-291.3, 291.5, 291.8, 291.81, 281.82, 291.89, 291.9, 303.00-303.93 or 305.00-305.03.

Demographic variables studied included age, gender, and race [14]. The comorbid psychiatric illnesses included mood disorders, anxiety disorders, and psychotic disorders, and among SUD we included tobacco, cannabis, cocaine/amphetamine (stimulants), and opioids. These comorbidities were identified using ICD-9 diagnosis codes [14].

Statistical analysis

We compared non-TBI and TBI cohorts using the bivariate analysis to evaluate the differences in demographics and comorbidities, and then in the separate bivariate analysis, we compared TBI inpatients with AUD versus without AUD. A multivariable logistic regression model adjusted for demographics, psychiatric comorbidities, and SUD was used to evaluate the odds ratio (OR) of AUD as a risk factor for TBI-related hospitalization. All statistical analyses set a priori at <0.01 were conducted on the Statistical Package for the Social Sciences (SPSS) version 25 (IBM Corp., Armonk, NY).

Ethical approval

Individual identifiers were used to protect the patient identity and other clinical information. The use of NIS under the HCUP does not require approval from the institutional review board as the NIS is a publicly available de-identified database [13].

## Results

The majority of the TBI inpatients were adolescents (12-18 years, 82.2%) and had higher odds (OR 1.46, 95% CI 1.43-1.49) for hospitalization for TBI compared to children under 11 years. TBI was prevalent in males (71.2% vs. 28.8% females) and they had three times higher odds (95% CI 3.14-3.26) for TBI-related hospitalization compared to females. TBI inpatients were seen majorly in whites (59.2%) and there was a statistically significant difference race-wise with minorities at lower odds for TBI-related hospitalization.

Among psychiatric comorbidities, mood (4.1%) and anxiety (2.2%) disorders were prevalent in TBI inpatients, and when compared with non-TBI cohort, TBI inpatients have lower odds of association with psychiatric comorbidities. Among substance use disorders (SUDs), alcohol and tobacco use (4.4% each), and cannabis use (3.5%) were prevalent. SUD did not have a positive association with TBI-related hospitalization. AUD was associated with higher odds (OR 3.5, 95% CI 3.35-3.67) of TBI-related hospitalization in the logistic regression model after adjusting for demographics, psychiatric comorbidities, and SUD as shown in Table 1.

**Table 1:** Predictors for traumatic brain injury-related hospitalization TBI, traumatic brain injury

Variables	Non-TBI	TBI	Odds ratio	95% CI	P-value
Total inpatients	3,763,575	61,948	-	-	-
Age groups, %					
8-11 years	20.3	17.8	Reference		
12-18 years	79.7	82.2	1.46	1.43-1.49	< 0.01
Sex, %					
Male	43.4	71.2	3.19	3.14-3.26	< 0.01
Female	56.6	28.8	Reference		
Race, %					
Caucasian	51.6	59.2	Reference		
African American	20.1	14.3	0.62	0.61-0.64	< 0.01
Hispanic	20.8	18.8	0.79	0.77-0.81	< 0.01
Other	7.5	7.8	0.89	0.87-0.92	< 0.01
Psychiatric comorbidities, in %					
No comorbidities	-	-	Reference		
Anxiety disorders	8.8	2.2	0.28	0.27-0.29	< 0.01
Mood disorders	8.5	4.1	0.52	0.50-0.55	< 0.01
Psychotic disorders	0.9	0.6	0.72	0.64-0.81	< 0.01
Substance use disorders, in %					
Alcohol	1.8	4.4	3.50	3.35-3.67	< 0.01
Tobacco	4.0	4.4	0.98	0.94-1.02	0.318
Cannabis	4.2	3.5	0.59	0.57-0.63	< 0.01
Opioid	0.6	0.2	0.35	0.29-0.42	< 0.01
Stimulants	0.6	0.5	0.85	0.75-0.96	0.01

TBI inpatients with AUD were majorly adolescents (96.4%) who had four times higher odds (95% CI 3.46-5.21) compared to children under 11 years. AUD was prevalent in males (77.2%) and they had higher odds (OR 1.17, 95% CI 1.06-1.29) than females. Though AUD was prevalent in Caucasians (53.3%), yet compared with them, hispanics had 1.9 times higher odds (95% CI 1.72-2.09) for AUD.

Statistically there was no significant difference for anxiety and psychotic disorders by comorbid AUD. TBI inpatients with AUD had higher odds (OR 1.62, 95% CI 1.37-1.92) for mood disorders. Among SUD, TBI inpatients with AUD had higher odds for comorbid stimulants (OR 5.11, 95% CI 3.85-6.77), cannabis (OR 4.69, 95% CI 4.12-5.34), and tobacco (OR 3.77, 95% CI 3.34-4.27) as shown in Table 2.

**Table 2:** Characteristics of traumatic brain injury inpatients with comorbid alcohol use disorder AUD, alcohol use disorder

Variables	Non-AUD	AUD	Odds ratio	95% CI	P-value
Total inpatients	59,206	2,644	-	-	-
Age groups, %					
8-11 years	18.5	3.6	Reference		
12-18 years	81.5	96.4	4.24	3.46-5.21	< 0.01
Sex, %					
Male	70.9	77.2	1.17	1.06-1.29	< 0.01
Female	29.1	22.8	Reference		
Race, %					
Caucasian	59.4	53.3	Reference		
African American	14.7	6.0	0.41	0.35-0.49	< 0.01
Hispanic	18.2	31.3	1.89	1.72-2.09	< 0.01
Other	7.7	9.3	1.37	1.18-1.59	< 0.01
Psychiatric comorbidities, in %					
No comorbidities	-	-	Reference		
Anxiety disorders	2.2	3.2	0.71	0.54-0.93	0.014
Mood disorders	3.9	8.5	1.62	1.37-1.92	< 0.01
Psychotic disorders	0.5	1.3	1.14	0.75-1.73	0.552
Substance use disorders, in %					
Tobacco	3.7	20.5	3.77	3.34-4.27	< 0.01
Cannabis	2.6	21.4	4.69	4.12-5.34	< 0.01
Opioid	0.2	0.4	0.35	0.16-0.77	0.01
Stimulants	0.3	4.3	5.11	3.85-6.77	< 0.01

## Discussion

The results of our large sample cross-sectional nationwide study found that AUD increases the risk for TBI-related hospitalization in the children and adolescent population by 3.5 times. AUD is an independent risk factor for TBI as the regression model was adjusted for potential confounders including SUD and psychiatric comorbidities.

TBI hospitalizations are more common in boys compared to girls among adolescents. This is probably due to an increased incidence of TBI due to MVA and/or intentional assault, which is common among boys [15]. In our study, adolescents (increased by 1.5 times) and males (increased by three times) had higher odds for TBI-related hospitalization. TBI is more common in blacks compared to other races due to the fact that high violence rates are seen in black teens [16]. Black children are significantly less likely to have ED visits after a head injury, and less likely to be diagnosed with brain concussion compared to whites [17]. According to a study on children and adolescent hospitalizations, most children with TBI are male and white [18]. This explains a higher prevalence of TBI-related hospitalization in whites compared to blacks and other races in our study.

Depression is common in post-TBI patients due to difficulty coping with the loss in the individual’s life [19]. MDD occurs in 10%-25% of post-TBI children [20]. TBI causes disruption in the neuronal circuit, which causes disruptions of the neurotransmitter circuits in serotonin, norepinephrine, dopamine, and acetylcholine leading to a higher risk of anxiety and mood disorders [21]. In our study, none of the psychiatric comorbidities were associated with increasing the risk of TBI-related hospitalization.

Adolescents are at high risk of addiction due to the continued brain and/or grey matter maturation and neurodevelopmental changes that take place in the frontal cortical and the subcortical monoaminergic systems. These changes explain the adolescents impulsive and novelty-seeking behavior traits [22]. TBI causes disruption to the developing nervous system, which increases the vulnerability to AUD and SUD [23]. TBI increases the risk of addiction by reducing the cognitive ability to perceive negative consequences and increases the perception of gains perceived by drinking and substance abuse [23]. A study by Patel et al. found that cannabis abuse among youth increases the risk of intracranial injury by 1.3 times [24]. Binge alcohol drinking is also a risk factor for TBI due to falls and assaults and MVA. After controlling for demographic confounders, psychiatric comorbidities, and other SUD, AUD was associated with statistically significant increased risk for TBI-related hospitalization.

AUD is more common in boys compared to girls, due to biological and social reasons. The increase in dopamine in the ventral striatum, more specifically nucleus accumbens, in boys is responsible for pleasure, reinforcement, and development of SUD [25]. Both sex face different social reinforcement and punishment attitudes regarding alcohol use. Alcohol use for boys is perceived as a sign of masculinity and independence in certain cultures, whereas among girls, it is expected to embrace the cultural stereotypical female characteristics like virtue and emotionality, consequently, they consume less alcohol [26]. In our TBI inpatients, AUD was seen majorly in males (77%) and adolescents (increased by four times compared to children <11 years).

Among those who drink alcohol, hispanics are at higher risk of AUD compared to non-hispanic whites [27]. This correlates with our study findings, as 31.3% hispanics had about two times higher odds for AUD than 53.3% whites. Alcohol, cannabis, and tobacco are the most common substances abused among adolescents in the United States, with prevalence rates 24%, 13%, 13%, respectively [28]. The co-occurrence of cannabis and tobacco use with alcohol is very common in adolescents. About 75% of American teens who use cannabis also use alcohol. The use of more than one substance increases the likelihood of more than one SUD [29]. According to the substance abuse and mental health services administration (SAMSHA), 53% of adolescents who are binge drinking, reported last month tobacco use, and 30% of binge drinkers and 56% of tobacco users reported cannabis use in the same period [28]. In our study, we found that TBI inpatients with AUD had four to five times higher odds for comorbid tobacco and cannabis use disorders. Alcohol and methamphetamine are frequently used together, and about 77% of those who have comorbid methamphetamine use disorders also meet the criteria for AUD. AUD increases the chances of methamphetamine use by four-folds for an increased euphoric effect [30]. This could be the possible reason behind TBI inpatients with AUD having five times higher odds for abusing stimulants.

An important limitation of our study is that the NIS data lack individual-level clinical information and the cross-sectional nature of the study prevents from confirming the causal relationship between TBI and AUD in hospitalized adolescents. But, using these national data, we were able to confirm a population-based association of AUD and TBI and an increase in hospitalizations in adolescents after controlling for possible confounder.

## Conclusions

AUD is an independent risk factor for TBI-related hospitalization with an increased risk of 50% in the children and adolescent population compared to non-alcohol users. TBI inpatients with AUD are prevalent in white, and male adolescents. These at-risk populations are also at higher risk of comorbid mood disorders and increased substance use including stimulants, cannabis, and tobacco. Alcohol screening and support groups, and behavioral health education programs should be provided to post-TBI patients with AUD as persistent alcohol use problems leads to cognitive deficits, worsening memory, learning abilities, and processing speed. They also have less tolerance to alcohol and more liability to falls, seizures, and subsequent TBI with low blood alcohol levels.
